# GOurmet: A tool for quantitative comparison and visualization of gene expression profiles based on gene ontology (GO) distributions

**DOI:** 10.1186/1471-2105-7-151

**Published:** 2006-03-17

**Authors:** Jason M Doherty, Lynn K Carmichael, Jason C Mills

**Affiliations:** 1 Department of Pathology and Immunology, Washington University School of Medicine, St. Louis, MO 63110, USA; 2 Departments of Molecular Biology and Pharmacology, Washington University School of Medicine, St. Louis, MO 63110, USA; 3 The Genome Sequencing Center, Washington University School of Medicine, St. Louis, MO 63110, USA

## Abstract

**Background:**

The ever-expanding population of gene expression profiles (EPs) from specified cells and tissues under a variety of experimental conditions is an important but difficult resource for investigators to utilize effectively. Software tools have been recently developed to use the distribution of gene ontology (GO) terms associated with the genes in an EP to identify specific biological functions or processes that are over- or under-represented in that EP relative to other EPs. Additionally, it is possible to use the distribution of GO terms inherent to each EP to relate that EP as a whole to other EPs. Because GO term annotation is organized in a tree-like cascade of variable granularity, this approach allows the user to relate (*e.g*., by hierarchical clustering) EPs of varying length and from different platforms (*e.g*., GeneChip, SAGE, EST library).

**Results:**

Here we present GOurmet, a software package that calculates the distribution of GO terms represented by the genes in an individual expression profile (EP), clusters multiple EPs based on these integrated GO term distributions, and provides users several tools to visualize and compare EPs. GOurmet is particularly useful in meta-analysis to examine EPs of specified cell types (*e.g*., tissue-specific stem cells) that are obtained through different experimental procedures. GOurmet also introduces a new tool, the Targetoid plot, which allows users to dynamically render the multi-dimensional relationships among individual elements in any clustering analysis. The Targetoid plotting tool allows users to select any element as the center of the plot, and the program will then represent all other elements in the cluster as a function of similarity to the selected central element.

**Conclusion:**

GOurmet is a user-friendly, GUI-based software package that greatly facilitates analysis of results generated by multiple EPs. The clustering analysis features a dynamic targetoid plot that is generalizable for use with any clustering application.

## Background

The advent of platforms for generating gene expression data for thousands of genes in a single experiment, has led to dozens of software packages to analyze the results of these experiments [1,2]. A fundamental need of biologists who generate gene expression profiles is to simultaneously compare multiple gene expression datasets to one another. Accordingly, many existing software packages allow such comparisons. For example, Melton and co-workers used dChip [3,4] to show that their gene expression profiles of embryonic, neural, and hematopoietic stem cells were more similar to each other than to profiles, generated in the same lab, of differentiated populations springing from those cells [5]. Lemischka and co-workers used different comparison tools to come to the same conclusion about their own profiles of essentially the same stem cells [6].

Most software tools currently available for comparison of multiple gene expression datasets work optimally when the profiles being compared are generated on the same platform and in the same lab [7]. Attempts at meta-analysis using data produced in multiple labs is typically fraught with technical difficulties and controversy, as has been the case when comparing the overlap between the Melton and Lemischka labs' stem cell expression data [8-10]. Such inter-lab difficulties are compounded when the platforms used to generate the expression profiles are even more divergent. For example, how would one begin to compare a gene expression profile generated by Affymetrix GeneChips (as in the examples above) to one generated by cloning and sequencing of a subtracted library of ESTs? Clearly, there is a need for "platform-independent" comparison software. Such software would aid biologists not only in direct analysis of their data with similar data in other labs but might also facilitate analysis of new datasets with respect to the thousands of previously prepared datasets, prepared using a variety of platforms, housed on various web sites throughout the world.

A few previous reports have described approaches to cross-platform analysis (see Moreau et al. [11] for review and, more recently, see ref. [12]). In general, these articles outline innovative methods for integrating expression intensity data for a given gene on one platform with intensity data for the same gene on another platform. Such techniques – along with a standardized system for sharing functional genomic data [13,14] – are critical when the aim is to follow changes in specific genes across multiple expression profiles. However, a gene expression profile also represents more than a list of its individual gene designations and their associated expression intensities: it can be viewed as the subset of the transcriptome that integrally specifies a given cell ("differentiates" it) from all others. At this level of interpretation, the intensity level of expression of individual genes in a cellular gene expression profile is not important. An individual gene is either a member of the subset of genes that defines a given cell type or population (*i.e*., is preferentially expressed in that cell relative to a reference cell population), or the gene is not a member of that subset. What is most important, from the biologist's point of view, is how all the genes that a given cell preferentially expresses organize themselves into higher order clusters of biological significance that can eventually shed light on that cell's unique function.

Accordingly, another approach for cross-platform comparison of gene expression profiles would involve integrating the biological functions/processes associated with the genes in each expression profile (independent of expression intensity of each gene) and comparing the profiles at the level of the biological significance of the member genes. We have previously used a preliminary version of this approach to show that the expression profile of gastric epithelial progenitors generated on Affymetrix Mu11K A & B Genechips [15] was more similar to a subtractive EST library of hematopoietic stem cells [16] than to various expression profiles of differentiated gastric epithelial cells by first classifying all the genes in each profile with their corresponding Gene Ontology (GO, [17]) terms and then comparing each profile to all the others based on the fractional representation of each GO terms within each list, where "fractional representation" of a GO term is defined as the number of genes annotated by a given GO term relative to the total number of genes in the expression profile. We have also used preliminary variations of this approach in other manuscripts [18,19].

Currently, there are several software packages that determine biological functions inherent to an expression profile, for example, by determining the distribution of GO terms associated with the member genes [20-23], and there are those which also identify GO terms that are over or under-represented in a comparison among different profiles [24]. GoMiner, for example, is a popular tool whose "High-Throughput" function calculates fractional representations of GO terms within a list of genes or across multiple lists of genes [25]. And output from GoMiner can be combined with a clustering/visualization tool such as CIMminer or Spotfire™ (Spotfire, Inc.), to cluster and relate fractional representation of individual GO terms across multiple gene lists. Here, we describe GOurmet, which, like some of the other tools, determines fractional representation of GO terms in multiple expression profiles and allows the user to identify over- and under-represented GO terms in comparisons among these profiles. But GOurmet is specifically designed to facilitate using GO terms in an alternative way: as a means of quantitatively relating entire expression profiles to each other. GOurmet also introduces the Targetoid Plot, a new way of dynamically re-rendering clustering data to allow users to determine pairwise distances between each element in the cluster.

## Implementation

**Figure 1:**
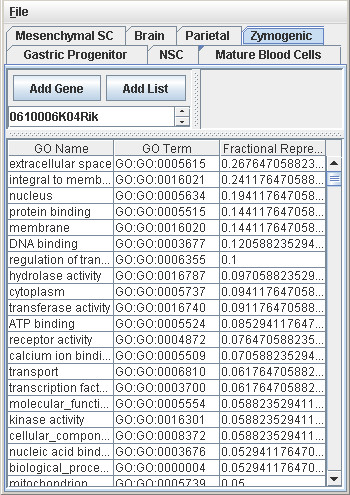
**The GOurmet Vocabulary program calculates and displays the fractional representation of every GO term in each inputted expression profile**. Depicted is a screen capture of the GUI showing the fractional representation of GO terms in a sample gene expression profile (in this case, the list of genes preferentially expressed in gastric zymogenic cells). Other profiles can be selected by clicking on the tabs. Results can be outputted in a multi-sheet MS Excel Workbook file (not depicted) for each expression profile, where one sheet summarizes the statistics of the annotation (number of genes in the profile, percent of those genes successfully annotated with GO terms, etc.), the second sheet lists every GO term associated with each gene, the third lists all the GO terms found in the profile, followed by the genes associated with each GO term. Results can also be output as tab-delimited Comparison files as input for GO Cartography.

**Figure 2:**
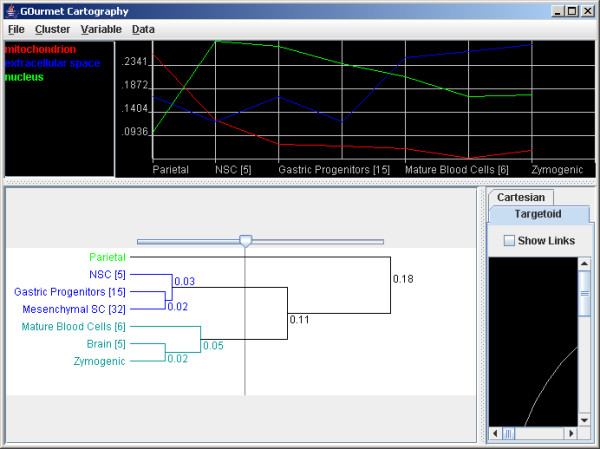
**The GOurmet Cartography dendrogram and wave plot windows**. *Bottom left *– the GOCART dendrogram plots all the expression profiles in a hierarchically clustered dendrogram using a modified Pearson's correlation to determine distances between clusters. A sliding bar allows the user to select how sub-clusters are colorized to visualize differences. The dendrogram can be exported as a jpeg. In the example, note how the three sample progenitor expression profiles (MeSC [32], NSC, and GEP) cluster together, and three of the four sample differentiated cell lineage expression profiles cluster together (mature blood, brain, and zymogenic cells [33]). Parietal cells, a highly specialized, differentiated, mitochondria-rich cell type cluster separately, well away from the other lineages. Numbers in brackets indicate reference from which each expression profile was acquired. *Top *– the GOCART wave diagram allows users to select GO terms (listed at the left of the window) to view their fractional representation among selected expression profiles (profiles to be viewed are selected from the dendrogram plot). Each GO term is automatically assigned a different color in the plot. In the example, note how much higher the fractional representation of the GO term "mitochondrion" (red line) is in parietal cells relative to the other sample expression profiles. The wave diagram can be output as a jpeg image.

**Figure 3:**
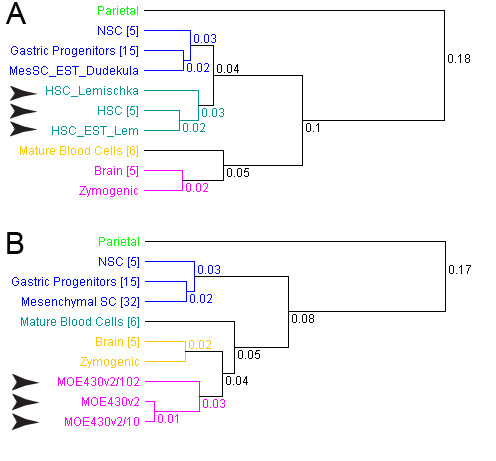
**GOurmet can cluster expression profiles from varying platforms, from different labs, and of different lengths**. A) Three different hematopoietic stem cell (HSC) expression profiles (one generated from a subtracted library [16], two from different GeneChips in different labs [5,6]) all cluster most closely with each other (marked by arrowheads) than with any of the other progenitor or differentiated cell profiles. B) A parent gene list composed of every annotated gene on the Moe430V2.0 GeneChip was reduced in length 10-fold by random exclusion of 9 in 10 genes and 100-fold by exclusion of 99 in 100 genes. The reduced length versions of the original list still cluster with the parent (marked by arrowheads). [32]

GOurmet is a software package that takes as input multiple gene lists/profiles from any large-scale gene expression method, computes the distribution of GO terms associated with the genes in each list and finally compares these expression profiles at the level of their inherent biological function, visualizing the results using clustering and other graphical formats, some familiar and some novel. GOurmet, written entirely in Java, has a simple architecture, consisting of two inter-related software packages: GOurmet Vocabulary and GOurmet Cartography. GOurmet Vocabulary is a simple tool to annotate by GO terms gene lists provided by the experimenter (Fig. 1). Similar to some other GO term analysis packages, this tool affords the user a quick assessment of biological value in the data that may have been obscured by the data's sheer size. GOurmet Cartography is a tool for semi-quantitatively relating and analyzing different gene expression profiles at the level of biological meaning (Fig. 2). GOurmet Cartography analyzes data in a way that is largely 1) independent of the platform used to generate the expression profile and 2) independent of the number of genes within each expression profile. Point (1) can be illustrated by showing that profiles of the same cell types, even when generated from multiple platforms and labs, still cluster together: *e.g*., HSCs cluster with other HSCs whether the HSC profiles are generated on GeneChips by different labs [5,6] or generated from a subtracted EST library [16] (Fig. 3A). We illustrate point (2) by showing that we can reduce a large expression profile by 10- and even 100-fold in length (by randomly eliminating 9 in 10 or 99 in 100 genes from the original list), and the reduced profiles, expressed in terms of GO distribution, still cluster more closely with the original, full length profile than with other profiles (Fig. 3B). The platform and list-length independence are largely the result of GOurmet's super-organizing gene lists into GO distributions. In the Gene Ontology system, gene products are classified by a tree-like cascade of GO terms, which themselves range from quite specific (*e.g*., "succinate dehydrogenase activity" applying to only a few genes) to broad (*e.g*., "nucleus", applying to up to a quarter of the genes in the transcriptome). Thus, even though gene expression profiles as short as a few hundred genes might be difficult to compare to much longer profiles at the level of specific, less frequent GO terms, they can still be meaningfully compared at the broad level (*i.e*., using the respective fractional representations of GO terms such as "nucleus" and "integral to membrane"). And, it should be noted a difference in distribution of such broad GO terms between two expression profiles has obvious biological relevance (*e.g*., cells with increased expression of genes localizing to the nucleus tend to be less differentiated). A caveat that should also be noted is that because GOurmet takes as input an expression profile in the form of a list of genes with no restrictions on length of the list or how the list was generated (what platform, what stringency for inclusion of genes, etc.), the program does not attempt to provide a useful assessment of error or statistical significance for profile clusters. In our empirical experience, however, expression profiles that are at least 400 genes long allow relation of profiles in a way that is biologically meaningful.

## Results and discussion

### GOurmet vocabulary

This component of the program suite accepts a tab-delimited text file comprising one to multiple lists of Unigene-standard gene symbols (each representing, for example, the expression profile of a given cell type) and translates each list into GO term distributions by querying a local MySQL database built from the latest GO term annotations downloaded from the Gene Ontology website [17] (Fig. 1). For example, to compare our list of genes that characterize gastric epithelial progenitors (GEP) to the list of genes that characterize mesenchymal stem cells (MeSCs), we prepare an Excel spreadsheet with "GEP" in the first cell, followed by all the gene symbols in the GEP profile and do the same for "MeSC" and all its associated symbols. GOurmet Vocabulary then queries the GO database to determine the GO terms associated with each gene symbol and computes the fractional representation of all the GO terms associated with the genes in the list. The program uses a GUI, where each profile is a tab that can be clicked on to show the fractional representation of each GO term (*i.e*., the GO term distribution) across the entire profile. Results can be output as multi-sheet Microsoft Excel workbook files or as "Comparison" files which are tab-delimited text files. The latter files serve as inputs for the adjoining GOurmet Cartography suite.

### GOurmet cartography

The other half of the suite, GOurmet Cartography (GOCART) is devoted to semi-quantitative analysis and comparison of GO profiles from GOurmet Vocabulary. Input is via "Comparison" files, which are the tab-delimited files output by GOurmet Vocabulary. A Comparison file from GOurmet Vocabulary comprises one to multiple expression profiles, each expressed in terms of relative frequencies of the GO terms associated with the transcripts in each profile. Users can also generate their own Comparison files, if they want to take advantage of the GOCART visualization tools but want to compare elements (such as expression profiles) based on variables other than GO term distributions (see below). The program allows unlimited additional Comparison files to be merged into the active workspace. Because GOCART also allows output of tab-delimited Comparison files, any additions (or deletions) to the expression profiles in the workspace can be saved. All profiles are automatically clustered (see below).

#### Dendrogram and wave plots

GOurmet Cartography employs a multi-window, user-friendly GUI. The dendrogram (left window in program, Fig. 2) is a graphical rendering of the results of relating the gene profiles created in Vocabulary. Relationships between each profile are calculated by means of multiple set-wise adapted Pearson's correlations between elements and then between clusters of elements. Distance (d) between two elements is calculated as:

d = |PC - 1|

A Pearson correlation of 1 equals a distance of 0, and a correlation of -1 equals a distance of 2. Adapting a Pearson's correlation in this fashion is a common way to produce a metric for quantifying similarities between elements of a cluster [26,27]. The program offers several algorithms for displaying distance between sub-clusters, including: Single-Link, Complete-Link and Average-Link. In single-link clustering, the distance between two sub-clusters is the distance between their closest elements. In complete-link clustering, it equals the distance between their farthest elements. In average-link clustering, it is the average distance between their elements. The program allows the user to take the average of multiple individual profiles and cluster the consensus profile as a separate entity (*e.g*., one can make a consensus "stem cell" profile based on the average of each of the individual stem cell GO distributions and see where the stem cell consensus clusters relative to other profiles).

The wave diagram (upper window, Fig. 2) is a feature that allows the user to view profiles selected in the dendrogram window in a wave guide or flowing plot format. It shows the fractional representation of a user-selected GO term or terms within each selected expression profile. By default, GO terms are listed in decreasing order of their weight in differentiating among selected profiles (though choosing the GO terms most similar among the selected profiles is also an option; see below). For example, if one wanted to know which single GO term was most responsible for differentiating a gene expression profile of the gastric parietal cell from that of the gastric epithelial progenitor, the program would return "Mitochondrion", and the fractional representation of that term would be plotted in the parietal cell and gastric epithelial progenitor profiles (note that the wave diagram in Fig. 2 shows how much more common the term "Mitochondrion" is in the parietal cell profile relative to all other profiles). The utility of this feature is evident for users who want to determine how their cell profile is similar to reference profiles. The fact that the term "Mitochondrion" is one of the most important for differentiating the parietal cell expression profile from all the other profiles is consistent with the known function of parietal cells as mitochondrion-rich energetic powerhouses [15,28]. GOCART determines the weight in establishing differences among expression profiles from the absolute standard deviation (SD) of the fractional representation of that GO term among the expression profiles selected. GOCART can also establish the GO terms that are most important, at the absolute level, in determining similarity (V_s_) between expression profiles. This is calculated as:

V_s _= (cluster_mean - total_mean) * (cluster_mean/total_mean), where "cluster mean" is the mean fractional representation of a given GO term among the expression profiles selected, and "total mean" is the fractional representation of that GO term across all the expression profiles in the current dendrogram window.

Furthermore, users can apply a standardizing expression profile to highlight GO terms that deviate substantially from a reference (*e.g*., the expression profile of every gene on a given Affymetrix GeneChip set). Here, GO terms with the highest *relative *weight in differentiating expression profiles are calculated as:

V_s _= SD * (cluster_mean/baseline), where "cluster mean" is the mean fractional representation of the given GO term in all selected expression profiles, and "baseline" is a single expression profile chosen by the user. In determining relative cluster similarity, this is rendered by:

V_s _= (cluster_mean - total_mean) * (cluster_mean/total_mean) * (cluster_mean/baseline), where variables are as above.

This feature allows users to highlight more specific GO terms (*i.e*., ones that have lower fractional representations within the expression profiles and have more specific biological meaning; *cf*. the specific GO term "glutamate receptor activity" to the much more general "integral to membrane"). GO terms such as these may be important for differentiating closely related profiles or may represent the specific types of genes a biologist would like to follow up.

#### Targetoid plot

**Figure 4:**
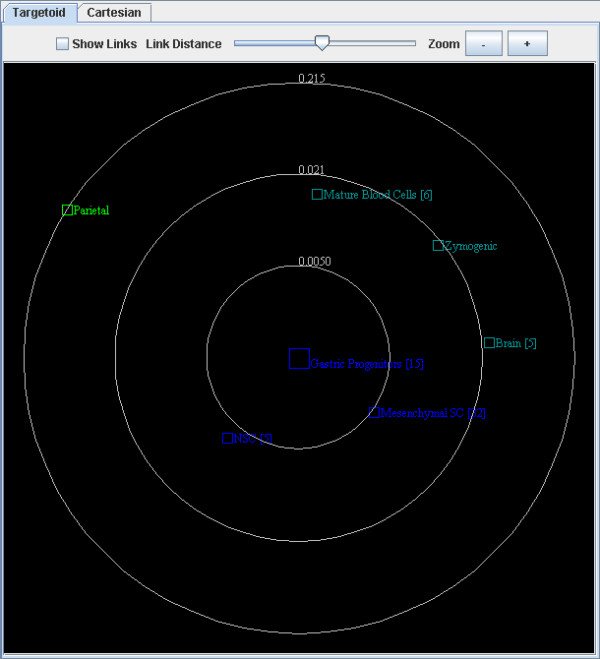
**The GOurmet Cartography Targetoid plotting window**. Depicted is a screen shot where the sample GEP profile has been chosen as a center element (center elements are selectable in the dendrogram window), and all other profiles radiate outward in direct inverse proportion to how similar they are to GEPs. Note how GEPs are more similar to the other two stem/progenitor profiles and more distant from all the profiles of differentiated cells. Relationships among non-center elements are only approximately reflective of their similarity and are depicted by how far apart they are from each other (θ angle between elements in this polar coordinate system). Any profile can be selected as the center and, by multiply selecting different profiles, users can reconstruct all the direct relationships among the various profiles. A sliding bar allows the user to focus on certain regions of the targetoid space (important for visualization when large numbers of profiles are compared at once and many similar ones cluster closely together); users can also adjust the axial scale. The targetoid plot can be output as a jpeg.

This window is a novel way of dynamically rendering the multi-dimensional relationships among all the elements in a clustering dataset. The standard dendrogram rendering of a clustering analysis allows users to see distances only between two elements within the same sub-cluster. For example, the dendrogram in Fig. 2 gives the Pearson's distance between gastric progenitors and MeSCs (0.02), but it gives only the distance between the *average *of the entire sub-cluster composed of MeSC and gastric progenitors to the NSC element (0.03). To calculate the distance between GEPs and NSCs, GOCART uses the Targetoid plot to compare these two profiles directly. The user can select one element (*e.g*., the GEP expression profile) as the center. Every other element is then plotted by the algorithm so that distance from the center is proportional to the similarity/difference between a given element and the central element, where similarity/difference is calculated using the Pearson correlation-derived formula. The algorithm also allows an approximate visualization of the similarity/difference among non-central elements (though the exact similarity/difference between each element and every other element cannot be depicted in this 2-dimensional plot for elements totaling greater than three). Non-center elements' positions are spread out in the radial dimension, so that the farther they are from each other, the less similar they are. The approach uses a rough heuristic for placing the elements, gradually adjusting their positions relative to each other, to achieve a "spread" of elements. The example in Fig. 4 shows that NSCs, MeSC, and GEPs are all quite similar, but MeSCs and GEPs are slightly more related to each other than to NSCs. The three differentiated cell lineage expression profiles cluster approximately together, and parietal cells are not related to any of the other elements. There is a zoom slider that allows the user to focus on certain regions of the targetoid plot.

#### Cartesian plot

**Figure 5:**
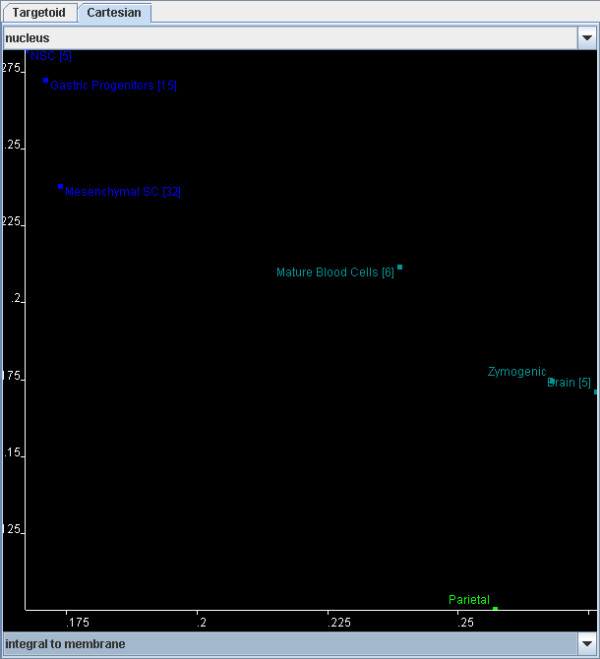
**The GOurmet Cartography Cartesian plotting window**. This window toggles with the Targetoid plot. Here, users can select one GO term as the y-axis and one as the x-axis. All the expression profiles are plotted according to their fractional representation of the selected GO terms. Note how the two selected GO terms ("nucleus" and "integral to membrane") distinguish expression profiles of stem/progenitor cells as a group from those of differentiated cells.

An additional window option (which toggles with the Targetoid plot) is the Scatter or Cartesian plot (Fig. 5). A user can select two individual GO terms in this window, and the program will plot how the various expression profiles relate to one another, using fractional representation of one GO term as the x-coordinate and that of the other as the y-coordinate. In the example of Fig. 5, the relationship between the GO terms "nucleus" and "integral to membrane" across the provided profiles shows how combinations of just two GO terms can help cluster broad categories of expression profiles (in this case, differentiating progenitor and differentiated cells).

#### Future refinements

The principal planned refinement to the software include increasing the number of clustering options (in addition to Pearson's correlation-based one currently available) and increasing the output options of GOurmet Vocabulary so that GO annotations can be added to an existing table of genes and gene annotations (*e.g*., one previously generated in Excel).

## Conclusion

GOurmet is a tool for comparing multiple expression profiles or gene lists independent of platform used to generate the expression profiles. The GOurmet Cartography package of the software also provides users several tools to visualize the results of such comparisons. The dendrogram tool allows the user a familiar way to visualize, at a glance, hierarchical relationships among multiple expression profiles. The new Targetoid graphing tool then allows the user to go beyond the standard hierarchical display by dynamically redrawing the data as a constellation with the user's choice of a center point. By dynamically redrawing the constellation with several different central points, users can extract the most salient aspect of the multi-dimensional data in a typical clustering comparison. The wave and Cartesian plot tools of GOCART allow the user to identify the principal GO terms that are most responsible for the way each expression profile clusters relative to the others. Because GO terms define cellular locations, functions and processes, these tools allow users to quickly determine what the biological significance of the clustering pattern is, thereby generating hypotheses for future testing.

We, and our collaborators, have used GOurmet to compare numerous stem/progenitor profiles to identify biological functions and processes associated with stemness and with differentiation (manuscript in preparation). We have also used GOurmet to compare expression profiles inter-species. Specifically, we have found that expression profiles of mouse tissues/cells cluster with human expression profiles for the same cells, largely ignoring species boundaries (data not shown). It should be mentioned, though, that GOurmet would be most useful for species whose genomes have already largely been annotated with GO terms (*e.g*., mouse and human).

We have also used GOCART with variables other than GO terms and elements other than expression profiles for better visualization of clustering results (for example, we have clustered the biological literature, where PubMed abstracts are the elements/profiles and the distribution of biological keywords in each abstract are the variables; others have shown how biomedical articles lend themselves to a clustering, meta-analytic approach [29]).

GOurmet is modular and is open source in Java, so individual tools such as the targetoid plotting algorithm could easily be imported into other packages (see below). We enthusiastically support such endeavors.

## Availability and requirements

First, note that the GOurmet Cartography software is designed to parse tab-delimited files from GOurmet Vocabulary as input, so users can easily create their own Comparison files for input. Thus, the analysis would not have to be limited to GO fractional representations but could be genes with corresponding intensities (or PubMed articles and keywords; see above). We think our multi-dimensional/multi-window rendering of clustering results should be useful in any number of classification and comparison applications.

GOurmet is written in Java and is open-source under the BSD license. The program requires that the user have installed the Java Runtime Environment 1.5.0 or higher. GOurmet is fully OS-independent as assembled under the Java Virtual Machine. Any OS that has the appropriate JRE installed (Microsoft Windows, Mac OS X, Linux, etc) may run it. GOurmet currently has many functions available only through right mouse click; on the Mac OS X, [Cmd-Click] will bring up the right button menu on the single-button Mac mouse.

GOurmet requires access to a MySQL database populated with Gene Ontology annotations released form the Gene Ontology Consortium. While GOurmet is by default configured to anonymously attach to our database at Washington University, it is highly encouraged for users to establish a local MySQL GO database and point the GO Vocabulary program to this local instance. Instructions for doing this are available on the web site. MySQL is freely available [30], as is the GO database [17].

Download and other information (such as sample input and documentation) are available at our website [31].

Contact: 

## List of abbreviations

SAGE – Serial Analysis of Gene Expression

EST – Expressed Sequence Tags

GO – Gene Ontology

HSCs – Hematopoietic Stem Cells

GEPs – Gastric Epithelial Progenitor Cells

NSCs – Neural Stem Cells

GOCART – GOurmet Cartography

GUI – Graphical User Interface

SD – Standard Deviation

## Authors' contributions

JMD extensively beta-tested the software, wrote the documentation, designed/implemented the web-page, and co-wrote the manuscript. LKC wrote the Java code, co-designed the targetoid graphing approach and designed the targetoid pseudo multidimensional display algorithm. JCM beta-tested the software, implemented the original concept in MS Visual Basic, co-designed the targetoid graphing approach, and co-wrote the manuscript.

## References

[B1] Mills JC, Roth KA, Cagan RL, Gordon JI (2001). DNA microarrays and beyond: completing the journey from tissue to cell. Nat Cell Biol.

[B2] Dudoit S, Gentleman RC, Quackenbush J (2003). Open source software for the analysis of microarray data. Biotechniques.

[B3] Zhong S, Li C, Wong WH (2003). ChipInfo: Software for extracting gene annotation and gene ontology information for microarray analysis. Nucleic Acids Res.

[B4] Li C WWH, Parmigiani G GESIRAZSL (2003). DNA-Chip Analyzer (dChip). The analysis of gene expression data: methods and software.

[B5] Ramalho-Santos M, Yoon S, Matsuzaki Y, Mulligan RC, Melton DA (2002). "Stemness": transcriptional profiling of embryonic and adult stem cells. Science.

[B6] Ivanova NB, Dimos JT, Schaniel C, Hackney JA, Moore KA, Lemischka IR (2002). A stem cell molecular signature. Science.

[B7] Bammler T, Beyer RP, Bhattacharya S, Boorman GA, Boyles A, Bradford BU, Bumgarner RE, Bushel PR, Chaturvedi K, Choi D, Cunningham ML, Deng S, Dressman HK, Fannin RD, Farin FM, Freedman JH, Fry RC, Harper A, Humble MC, Hurban P, Kavanagh TJ, Kaufmann WK, Kerr KF, Jing L, Lapidus JA, Lasarev MR, Li J, Li YJ, Lobenhofer EK, Lu X, Malek RL, Milton S, Nagalla SR, O'Malley J P, Palmer VS, Pattee P, Paules RS, Perou CM, Phillips K, Qin LX, Qiu Y, Quigley SD, Rodland M, Rusyn I, Samson LD, Schwartz DA, Shi Y, Shin JL, Sieber SO, Slifer S, Speer MC, Spencer PS, Sproles DI, Swenberg JA, Suk WA, Sullivan RC, Tian R, Tennant RW, Todd SA, Tucker CJ, Van Houten B, Weis BK, Xuan S, Zarbl H (2005). Standardizing global gene expression analysis between laboratories and across platforms. Nat Methods.

[B8] Fortunel NO, Otu HH, Ng HH, Chen J, Mu X, Chevassut T, Li X, Joseph M, Bailey C, Hatzfeld JA, Hatzfeld A, Usta F, Vega VB, Long PM, Libermann TA, Lim B (2003). Comment on " 'Stemness': transcriptional profiling of embryonic and adult stem cells" and "a stem cell molecular signature" (I). Science.

[B9] Evsikov AV, Solter D (2003). Comment on " 'Stemness': transcriptional profiling of embryonic and adult stem cells" and "a stem cell molecular signature" (II). Science.

[B10] Vogel G (2003). Stem cells. 'Stemness' genes still elusive. Science.

[B11] Moreau Y, Aerts S, De Moor B, De Strooper B, Dabrowski M (2003). Comparison and meta-analysis of microarray data: from the bench to the computer desk. Trends Genet.

[B12] Stevens JR, Doerge RW (2005). Combining Affymetrix microarray results. BMC Bioinformatics.

[B13] Brazma A, Hingamp P, Quackenbush J, Sherlock G, Spellman P, Stoeckert C, Aach J, Ansorge W, Ball CA, Causton HC, Gaasterland T, Glenisson P, Holstege FC, Kim IF, Markowitz V, Matese JC, Parkinson H, Robinson A, Sarkans U, Schulze-Kremer S, Stewart J, Taylor R, Vilo J, Vingron M (2001). Minimum information about a microarray experiment (MIAME)-toward standards for microarray data. Nat Genet.

[B14] Stoeckert CJJ, Causton HC, Ball CA (2002). Microarray databases: standards and ontologies. Nat Genet.

[B15] Mills JC, Andersson N, Hong CV, Stappenbeck TS, Gordon JI (2002). Molecular characterization of mouse gastric epithelial progenitor cells. Proc Natl Acad Sci U S A.

[B16] Phillips RL, Ernst RE, Brunk B, Ivanova N, Mahan MA, Deanehan JK, Moore KA, Overton GC, Lemischka IR (2000). The genetic program of hematopoietic stem cells. Science.

[B17] the Gene Ontology. http://www.geneontology.org.

[B18] Pull SL, Doherty JM, Mills JC, Gordon JI, Stappenbeck TS (2005). Activated macrophages are an adaptive element of the colonic epithelial progenitor niche necessary for regenerative responses to injury. Proc Natl Acad Sci U S A.

[B19] Stappenbeck TS, Mills JC, Gordon JI (2003). Molecular features of adult mouse small intestinal epithelial progenitors. Proc Natl Acad Sci U S A.

[B20] Cheng J, Sun S, Tracy A, Hubbell E, Morris J, Valmeekam V, Kimbrough A, Cline MS, Liu G, Shigeta R, Kulp D, Siani-Rose MA (2004). NetAffx Gene Ontology Mining Tool: a visual approach for microarray data analysis. Bioinformatics.

[B21] Boyle EI, Weng S, Gollub J, Jin H, Botstein D, Cherry JM, Sherlock G (2004). GO::TermFinder--open source software for accessing Gene Ontology information and finding significantly enriched Gene Ontology terms associated with a list of genes. Bioinformatics.

[B22] Khan S, Situ G, Decker K, Schmidt CJ (2003). GoFigure: automated Gene Ontology annotation. Bioinformatics.

[B23] Beissbarth T, Speed TP (2004). GOstat: find statistically overrepresented Gene Ontologies within a group of genes. Bioinformatics.

[B24] Zhang B, Schmoyer D, Kirov S, Snoddy J (2004). GOTree Machine (GOTM): a web-based platform for interpreting sets of interesting genes using Gene Ontology hierarchies. BMC Bioinformatics.

[B25] Zeeberg BR, Feng W, Wang G, Wang MD, Fojo AT, Sunshine M, Narasimhan S, Kane DW, Reinhold WC, Lababidi S, Bussey KJ, Riss J, Barrett JC, Weinstein JN (2003). GoMiner: a resource for biological interpretation of genomic and proteomic data. Genome Biol.

[B26] Ben-Dor A, Shamir R, Yakhini Z (1999). Clustering gene expression patterns. J Comput Biol.

[B27] Claverie JM (1999). Computational methods for the identification of differential and coordinated gene expression. Hum Mol Genet.

[B28] Mills JC, Syder AJ, Hong CV, Guruge JL, Raaii F, Gordon JI (2001). A molecular profile of the mouse gastric parietal cell with and without exposure to Helicobacter pylori. Proc Natl Acad Sci U S A.

[B29] Glenisson P, Coessens B, Van Vooren S, Mathys J, Moreau Y, De Moor B (2004). TXTGate: profiling gene groups with text-based information. Genome Biol.

[B30] MySQL AB ::  The world's most popular open source database. http://www.mysql.com/.

[B31] GOurmet - Gene Ontology for the refined palette. http://gutsc.wustl.edu/GOurmet/.

[B32] Sharov AA, Piao Y, Matoba R, Dudekula DB, Qian Y, VanBuren V, Falco G, Martin PR, Stagg CA, Bassey UC, Wang Y, Carter MG, Hamatani T, Aiba K, Akutsu H, Sharova L, Tanaka TS, Kimber WL, Yoshikawa T, Jaradat SA, Pantano S, Nagaraja R, Boheler KR, Taub D, Hodes RJ, Longo DL, Schlessinger D, Keller J, Klotz E, Kelsoe G, Umezawa A, Vescovi AL, Rossant J, Kunath T, Hogan BL, Curci A, D'Urso M, Kelso J, Hide W, Ko MS (2003). Transcriptome analysis of mouse stem cells and early embryos. PLoS Biol.

[B33] Mills JC, Andersson N, Stappenbeck TS, Chen CC, Gordon JI (2003). Molecular characterization of mouse gastric zymogenic cells. J Biol Chem.

